# Methodology for Registration of Shrinkage Tumors in Head-and-Neck CT Studies

**DOI:** 10.1155/2015/265497

**Published:** 2015-05-18

**Authors:** Jianhua Wang, Jianrong Dai, Yongjie Jing, Yanan Huo, Tianye Niu

**Affiliations:** ^1^Department of Radiation Oncology, Ningbo Treatment Center, Ningbo Lihuili Hospital, Ningbo, Zhejiang 315000, China; ^2^Department of Radiation Oncology, Cancer Institute (Hospital), Chinese Academy of Medical Sciences, Peking Union Medical College, Beijing 100021, China; ^3^Key Laboratory of Particle and Radiation Imaging of Chinese Ministry of Education, Department of Engineering Physics, Tsinghua University, Beijing 100084, China; ^4^The Second Affiliated Hospital of Zhejiang University School of Medicine, Hangzhou, Zhejiang 310009, China; ^5^Sir Run Run Shaw Hospital, Institute of Translational Medicine, Zhejiang University, Hangzhou, Zhejiang 310016, China

## Abstract

Tumor shrinkage occurs in many patients undergoing radiotherapy for head-and-neck (H&N) cancer. However, one-to-one correspondence is not always available between voxels of two image sets. This makes intensity-based deformable registration difficult and inaccurate. In this paper, we describe a novel method to increase the performance of the registration in presence of tumor shrinkage. The method combines an image modification procedure and a fast symmetric Demons algorithm to register CT images acquired at planning and posttreatment fractions. The image modification procedure modifies the image intensities of the primary tumor by calculating tumor cell survival rate using the linear quadratic (LQ) model according to the dose delivered to the tumor. A scale operation is used to deal with uncertainties in biological parameters. The method was tested in 10 patients with nasopharyngeal cancer (NPC). Registration accuracy was improved compared with that achieved using the symmetric Demons algorithm. The average Dice similarity coefficient (DSC) increased by 21%. This novel method is suitable for H&N adaptive radiation therapy.

## 1. Introduction

Image registration is becoming a key tool in modern radiation therapy. In image-guided radiation therapy (IGRT), CT images acquired before each treatment are registered with planning CT images to verify patient position. In adaptive radiation therapy, similar registrations are needed to segment structures automatically and to evaluate the dose received in each fraction [1–3]. Rigid registrations are sufficient in situations where only rigid movements occur. However, deformable registrations are required in cases where structure deformation and/or tumor shrinkage occur.

The majority of patients with head-and-neck (H&N) cancer who undergo fractionated radiation therapy experience significant anatomic changes, such as tumors shrinkage, changes in overall body habitus, and weight loss [4–6]. Tumor shrinkage is mainly due to the death and destruction of cells killed by radiation. The loss of these cells causes tumor density and tumor volume to decrease. This means that physical one-to-one correspondence may no longer exist between voxels of the two image sets. This problem severely impairs the performance of an intensity-based deformable registration algorithm. The “Demons” family of algorithms is such a registration form, which can accurately account for anatomical changes at relatively low computational expense [7–10], and is a suitable choice for registration of H&N images [11].

The gas pocket mismatching problem in abdominal images also provides challenges for the intensity-based deformable image registration. Several methods have been used to overcome the problems associated with lack of correspondence and mismatched objects that occur with deformable registration of abdominal CT images [12–16]. In images of the pelvic regions, the issue of no correspondence is associated with the presence of bowel gas. Because the gas is not clinically important (the contents of the bowel need not be registered), the artificial gas [12, 13] or constant intensity masks [14, 15] were introduced to create “virtual” correspondence. This improved the registration accuracy for the surrounding tissues, such as the rectal wall or prostate, which were clinically important organs. However, in the case of tumor shrinkage, the tumor itself is the treatment target. We could not use the constant intensity masks (or artificial intensity pattern) in the tumor, where the noncorrespondence problem occurred. For this reason, the approach mentioned above is infeasible. To our knowledge, the issue of lack of correspondence induced by the radiation therapy has not been adequately described in the literature.

In this paper, we describe a novel method for dealing with the effects of radiation on tumor tissues in the H&N during deformable registration. The method involved modification of image intensities of the primary tumor by calculating tumor cell survival rate using the linear quadratic (LQ) model according to radiation dose delivered to the tumor [17].

## 2. Materials and Methods

### 2.1. Data Extraction

The study included 10 patients with nasopharyngeal cancer (NPC) who underwent intensity-modulated radiation therapy (IMRT) at Cancer Institute (Hospital) of Chinese Academy of Medical Sciences, China. Patients were immobilized by custom-made thermoplastic masks. Two CT image sets were acquired for each patient (before the start of treatment and at the completion of the treatment course) using a Philips Brilliance Big Bore 16-slice CT scanner. The image sets were composed of 3 mm thick slices; the matrix size of each slice was 512 × 512 and the pixel size was approximately 1 mm. The initial image sets were loaded into a Pinnacle version 8.2g system (Philips Medical Systems, Cleveland, OH) for treatment planning. To improve the registration speed, the number of slices for each image pair was reduced so that it just covered the whole primary tumor. According to our standard treatment protocol, the primary tumor was prescribed 70–72.6 Gy. The tumor volume was contoured by a single physician for all planning and posttreatment CTs. Contouring was undertaken to evaluate the registration results, rather than to help the deformation process.

The CT images, contours, and treatment plan doses for each patient were exported from Pinnacle using the DICOM RT protocol. The exported dose array was a 3D dose map. The dose voxel size was 4 × 4 × 4 mm^3^.

The study was approved by the local ethical committee, and informed consent was obtained from all patients.

### 2.2. Image Preparation

The posttreatment CT was used as the static image and the planning CT as the moving image for each image pair. This represented a worst-case scenario for tumor shrinkage to test our proposed algorithm. The couch region was manually selected in the transverse plane and the voxels CT numbers were set to that of air (i.e., −1000 HU). Voxels CT numbers below the empirically determined −500 HU were also set to −1000 HU. This was done to reduce the interference from nonuniformities outside the patient.

Both the planning CT images and the posttreatment CT images were cropped on the transverse plane, to restrict the regions of interest (ROI) to the head. The images were resampled and determined to have the same voxel dimensions of 2 × 2 × 3 mm^3^ using the nearest interpolation.

### 2.3. Rigid Registration

Rigid registration was performed in advance to improve the speed and accuracy of the deformable registration. A minimum threshold of around 500 HU was applied to the two images so only the bony structures of the skeleton remained. The rigid registration determined a translation that minimized the correlation coefficient between voxels in the two images. To evaluate the accuracy of the rigid registration, we created a set of simulated translations of patient CT images. Compared to the introduced (known) shifts, the mean residual shifts (error) were 1.1 mm, within the voxel size of the patient images. This rigid alignment provided the basic initialization for subsequent deformable registration.

### 2.4. Deformable Registration

The “Demons” algorithm is an image intensity-based, deformable registration method that is widely used in medical practice as it demands relatively low computational expense. A variant of this algorithm, proposed by Wang, was used in the present study [18].

In the original Demons algorithm, the displacement for each voxel is obtained using the spatial gradient of static target image intensities. The displacement field is then regularized using a Gaussian smoothing filter to suppress noise and preserve the geometric continuity of the moving image. This iterative process alternates between calculation of the displacement field and regularization. By introducing an “active force,” Wang et al. modified the standard Demons algorithm to obtain faster convergence and improve registration performance. The updated deformation field *D*_*n*_ for the current iteration was written as follows:

(1) Dn=Gσ∗+∇⃑m∇⃑I′2+k2Dn−1+I′−I   ·∇⃑S∇⃑I2+k2I−I′2     +∇⃑m∇⃑I′2+k2I−I′2,

where *G*_*σ*_ is the Gaussian kernel, ∗ denotes the convolution operator, and the width of the Gaussian kernel *σ* was fixed to 1. *D*_*n*−1_ is the deformation field at iteration *n* − 1. *I*′ is the intensity of the moving image and *I* is the intensity of the static image; the respective gradient images are denoted by ${\overset{⃑}{\nabla }}_{I^{\prime }}$ and ${\overset{⃑}{\nabla }}_{I}$. *k* is a normalization factor and we used *k* = 0.4.

### 2.5. Intensity-Modification Procedure (IMP)

In patients undergoing radiation therapy, including H&N cancer, it is important that target volumes are accurately registered. Due to the cells killed by radiation, the density and even the volume of primary tumor may decrease. Unless this one-to-one correspondence is addressed, a large registration error will occur. We thus proposed to change the image intensities for the target volume in the planning CT image (moving image) based on the linear quadratic (LQ) model. The intensity modification was only done for the planning tumor and all other voxels of planning CT remained unchanged.

**Figure 1:**
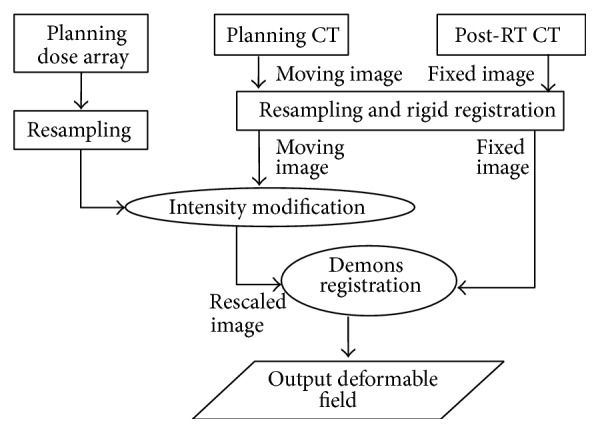
Method workflow consisting of the following procedures: rigid registration, intensity modification, intensity scale, and deformable registration.

The workflow of the proposed method is shown in Figure 1. We implemented all procedures described here using in-house program written in Matlab (version 7.1, MathWorks) software. Based on the planning dose array, we modified the image intensities within the primary tumor using the LQ model. The details of this procedure are described below.

#### 2.5.1. Data Import

The primary tumor volume contours as manually delineated during the routine treatment process were loaded into the planning CT images. The dose array was resampled to have the same spacing as the planning CT. The dose array and the planning CT were aligned according to their positions in the DICOM patient coordinate system. Thus, each element in the dose array represented the dose to be delivered to the corresponding voxel in the planning CT images.

#### 2.5.2. Image Intensities Modification

We calculated voxel intensity within the primary tumor using the LQ model. For every slice in the planning CT images, *I*_0_ represented the intensity of a voxel within the primary tumor. By definition, the relationship between *I*_0_ (CT numbers, expressed in Hounsfield units) and the corresponding linear coefficient *μ*_0_ was calculated as

(2) $$\begin{array}{c} I_{0}=\frac{1000\left(\mu_{0}-\mu_{w}\right)}{\mu_{w}}. \end{array}$$

Rearranging this gives

(3) $$\begin{array}{c} \mu_{0}=\frac{I_{0}\mu_{W}}{1000}+\mu_{W}, \end{array}$$

where *μ*_*W*_ is the attenuation coefficient of water (approximately 0.1928^−1^).

After the primary tumor cells in a given voxel were irradiated at dose *D*, the intensity value would decease to *I*_*S*_ (HU) with the corresponding attenuation coefficient *μ*_*S*_. *I*_*S*_ was converted to *μ*_*S*_ by the equation

(4) $$\begin{array}{c} \mu_{S}=\frac{I_{S}\mu_{W}}{1000}+\mu_{W}. \end{array}$$

As shown in (5), *μ*_*S*_ is proportional to the number of primary tumor cells *N*_*S*_ in a given voxel that survives irradiation, and *μ*_0_ is proportional to the number of cells in that voxel prior to irradiation *N*_0_:

(5) $$\begin{array}{c} \mu_{S}=\frac{\mu_{0}N_{S}}{N_{0}}=\mu_{0}\text{SF}, \end{array}$$

where, according to the LQ model, the survival fraction SF is given by

(6) $$\begin{array}{c} \text{SF}=\exp\left(-\alpha D\left(1+\frac{d}{\alpha /\beta }\right)\right). \end{array}$$

In this situation, cell proliferation is negligible. *d* and *D* are the dose per fraction and the total dose to the voxel, respectively. *α* and *β* are the LQ parameters. For the purposes of illustration, we assumed that *α* was 0.33 Gy^−1^ and *α*/*β* was 10 Gy [19–22]. Note that the result is not sensitive to the variation of *α* and *α*/*β* values as demonstrated in the result section. Using (3), (4), (5), and (6), we obtained

(7) $$\begin{array}{c} I_{S}=\left[\left(\frac{I_{0}\mu_{W}}{1000}+\mu_{W}\right)\exp\left(-\alpha D\left(1+\frac{d}{\alpha /\beta }\right)\right)-\mu_{W}\right]\times \frac{1000}{\mu_{W}}. \end{array}$$

#### 2.5.3. Voxel Intensity Scale

In the planning CT image, *S*_0_ was defined as the sum of voxel values within the primary tumor, and *S* denoted the sum of the voxel values within the corresponding region in the posttreatment CT image. The intensity value (*I*_*S*_) of the primary tumor voxel was scaled as follows:

(8) $$\begin{array}{c} I^{\prime }=\frac{I_{S}S}{S_{0}}. \end{array}$$

**Figure 2:**
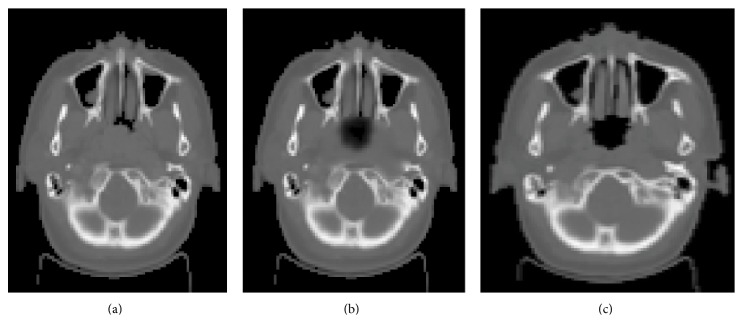
Example of intensity-modification procedure. (a) An axial slice of a planning image. (b) The same image after intensity modification. This modified planning image can be accurately registered using deformable image registration. (c) The corresponding slice from the posttreatment CT image.

Equations (7) and (8) were used to modify the intensity of every voxel of primary tumor in the planning CT images.  Figure 2 shows an axial slice of a planning image before and after image modification. This procedure was coupled with Wang's “Demons” algorithm to calculate the displacement field.

### 2.6. Evaluation

To quantitatively evaluate the performance of the proposed method, the Dice similarity coefficient (DSC) was calculated for the tumor. For two segmentations on the target images, given by the deformed contours using the computed motion fields and manual contours, respectively, their corresponding volumes were denoted by *V*_*d*_ and *V*_*m*_. The DSC was then defined as [23]

(9) $$\begin{array}{c} \text{DSC}=\frac{2V_{d}\cap V_{m}}{V_{d}+V_{m}}\times 100\%. \end{array}$$

DSC ranged between 0 and 100%. A DSC value of 0 indicated two completely uncorrelated images and a DSC value of 100% indicated a perfect match.

To assess the effectiveness of the proposed intensity modified procedure (IMP), we compared results of deformable image registration with and without intensity modification when all other parameters were set to the same. We also calculated the DSC for the tumor performed only with the rigid registration between these 10 pairs of CT images. The Wilcoxon signed-rank test was used to compare each method.

## 3. Results

### 3.1. Registration Example

**Figure 3:**
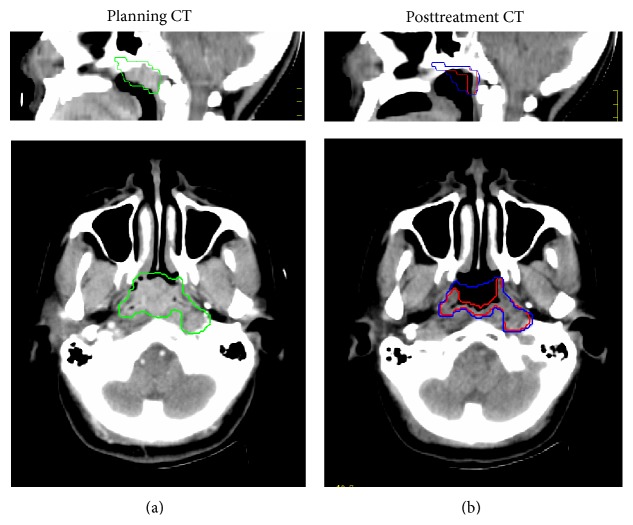
The CT images of one patient with the registered contours overlaid. (a) Planning CT in sagittal and axial slices and the manual contours overlaid. (b) The corresponding slices of posttreatment CT with the deformed contours overlaid. The red and blue contours represent the segmentation results with (red) and without (blue) the IMP.

To compare the effectiveness of the intensity modification, we performed deformable image registration in 10 pairs of head CT images with and without the intensity-modification procedure. An example is shown in Figure 3. The target volume change for this case was found to be 33% (from 15.59 cc to 10.47 cc). The left row of Figure 3 shows the planning CT in axial and sagittal views together with manually delineated tumor volume (green contours). The right row shows the posttreatment CT. Both sets of contours were overlaid in these images: the deformed contours without IMP (shown in blue) and the deformed contours with IMP (shown in red). As it can be seen in the figure, the deformed contours without using IMP clearly did not match with the reduced tumor target, due to the lack of one-to-one correspondence.

**Figure 4:**
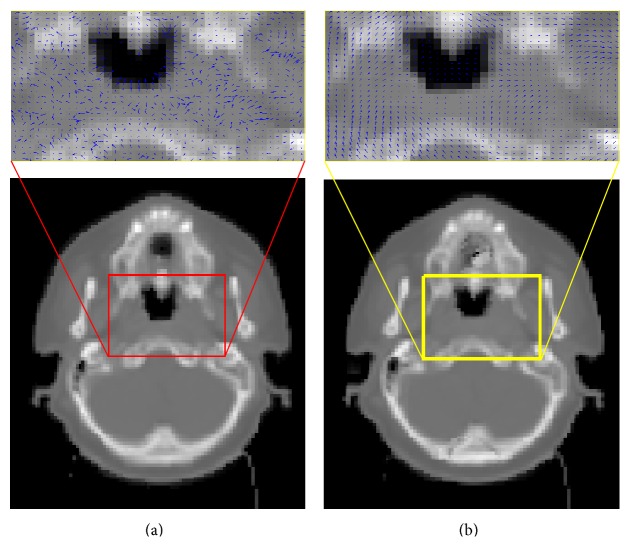
Displacement field calculated using the deformable registration algorithm overlaid on an axial slice. Displacement field: (a) computed without using the intensity-modification procedure (IMP) and (b) computed using the IMP.

Figure 4 shows the dense displacement fields with and without IMP. The arrows indicate the displacement in 3D but are projected as 2D images for display purposes. A vector field was used to assess the result and detect errors. It is evident from these displays that the displacement vectors (within the smaller region around the primary tumor where noticeable shrinkage occurs) are more chaotic, discontinuous, and abrupt, when IMP is not applied (Figure 4(a)). The voxels in this region exhibit unrealistic displacement due to the lack of correspondence. This is improved when the deformable registration is embedded with the intensity-modification procedure (Figure 4(b)).

### 3.2. Registration Statistics

The DSC values calculated for the tumor using rigid and deformable registration with and without IMP are summarized in Table 1. The Wilcoxon signed-rank test showed that there was little difference between the rigid registration and the deformable registration method without using the IMP method (*P* = 0.721). Nevertheless, there was a significant improvement when the IMP method was used over the rigid registration (*P* < 0.005) or the deformable registration method without using the IMP method (*P* < 0.005). The images with rigid registration had a mean overlap value of DSC = 76.3%. The mean value of the calculated DSC was 76.0% for deformable registration without IMP. The application of the IMP leads to a mean value of DSC = 92.0%. On average, the improvement in DSC was 21% with IMP in these 10 cases.

**Figure 5:**
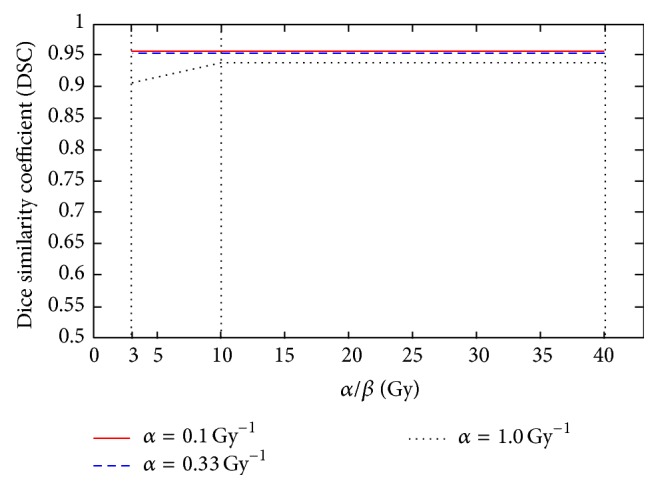
Sensitivity of the Dice similarity coefficient (DSC) with respect to *α* and *α*/*β* for patient 2.

In this study, hypotheses were made on the values of *α* and *α*/*β* ratio. The uncertainty in radiosensitivity parameters may affect the reliability of the outcome. Therefore, a sensitivity analysis was performed to quantify the uncertainty of DSC when different parameter combinations were applied in the study. A typical example is shown in Figure 5. We adjusted the key parameters (*α* and *α*/*β*) in a large range of possible values and provided the DSC results. It indicated that our method is not sensitive to the radiosensitivity parameters. For example, DSC slightly increased from 93.6% to 95.3%, even with more than one order of magnitude change on *α* and *α*/*β* values (*α* from 0.1 Gy^−1^ to 1 Gy^−1^ and *α*/*β* from 3 Gy to 40 Gy).

The computing time to perform the deformable registration varied depending on the number of registered image slices. For a typical planning and posttreatment CT pair, the run time for the full registration was approximately 15 min. These results were obtained on a Dell desktop PC with dual 2.13 GHz Intel Xeon processors and 2.25 GB of RAM.

As our focus was the accuracy of registration, we made no special effort to improve the speed of the registration process. However, time saving could be achieved by improving computer hardware such as GPU-based implementation of the Demons algorithm [24, 25].

## 4. Discussion

Previous studies have shown that nonrigid anatomical changes can occur in H&N during the course of fractionated radiation therapy [26]. The Demons algorithm has been shown to be a good choice for registration of H&N images [11]. However, the issue of primary tumor density change/volume shrinkage due to radiation treatment has not been addressed by the authors. In our experience, registration accuracy using this algorithm is reduced under these circumstances.

In the current study, we used a modification to the Demons algorithm which adjusted the intensities of the primary tumor voxels according to the LQ model. Qualitative and quantitative results demonstrated that the proposed method increased the performance of the registration in presence of tumor shrinkage.

The intensity-modification procedure used in this study is essentially a preprocessing method that can be used in conjunction with other intensity-based deformable registration algorithms. While the focus of the current work was to evaluate deformable registration in H&N region, the method may also be adapted to other anatomical regions where tumor shrinking occurs (e.g., in lung cancer). In lung cancer, noncoplanar beams may be used and more slices should be included along both the superior and inferior directions to the tumor.

The LQ model is generally effective in describing tumor response to radiation and is widely used in experimental and clinical radiobiology. So we chose this model to calculate the NPC cell killing by radiation. The cell killing process is a complicated biological process. However, this preliminary model could serve as a basis for more complex models to deal with the problem of tumor shrinkage. Considering the nonhomogeneous dose distributions in IMRT, we assumed that the primary tumor volume was composed of a series of subvolumes (voxels), each one receiving a homogeneous dose. In this setting, the basic LQ model may be used to estimate the NPC cell killing by radiation. We also recognize that further studies would be desirable to validate this assumption.

The radiation therapy parameters values used in our study were derived from the literature [19–22]. The ratio *α*/*β* was assumed to be 10 Gy, which is a nominal value for most tumors, and *α* was set at 0.33 Gy^−1^. Radiobiology parameters have a high degree of uncertainty caused by interpatient variations, tumor heterogeneity, and effects of hypoxia and chemotherapy. To deal with this uncertainty, we used a scale procedure within the LQ model to fix the potential problems caused by the patient and tumor specific biological parameters. The scale adjustments were based on information from pretreatment CT images. The sensitivity analysis showed that our method was not sensitive to the radiosensitivity parameters. It may also be possible to obtain biological information for individual patients noninvasively using functional imaging [27, 28].

In the formulation for the deformation field, we have chosen *σ* to be 1 and *k* to be 0.4 based on previous studies done by other investigators [7, 8, 11, 14, 18] and our own initial testing experience. The impacts of the *σ* and *k* parameters were not investigated thoroughly in this preliminary study. However, these specific values work well in most of our cases. Adjusting parameters by a trial-and-error method was also tested. No significant improvement was observed compared to the fixed values. In addition, our emphasis is on the comparison of the various schemes and not the final performance. Therefore, we used the same set of parameters for all experiments, without multiresolution adjustments.

**Figure 6:**
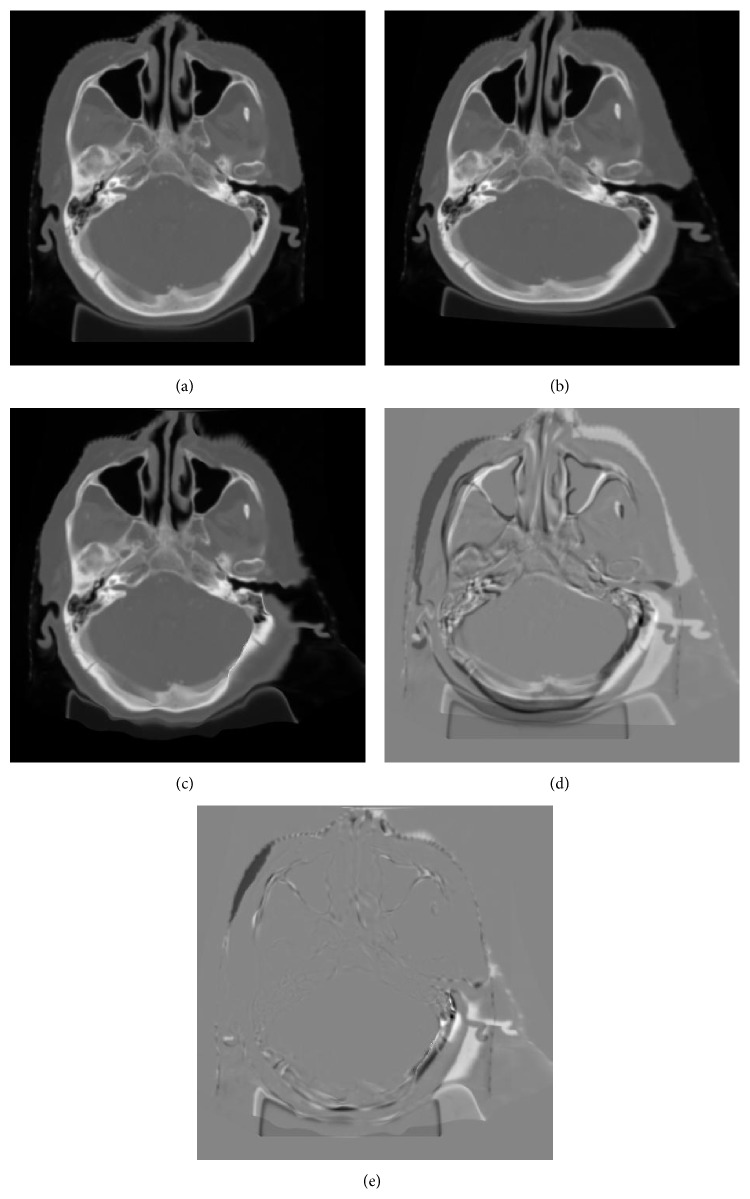
(a) Original image; (b) original image transformed by a “polynomial” transformation; (c) deformed image derived from (a) using the Demons algorithm; (d) difference image of (b) and (a); (e) difference image of (b) and (c).

Validation of deformable image registration remains a difficult task due to the lack of the ground truth. Our deformable registration algorithm was validated by simulating the deformation on patient CT image. We applied a 2nd-order polynomial transformation to the original H&N image (Figure 6(a)) and deformed its shape by more than 5 mm on average (Figure 6(b)). Our algorithm automatically generated the deformation field and deformed the original image to the mathematically transformed one (Figure 6(c)). The difference images (Figures 6(d) and 6(e)) were, respectively, Figures 6(b)-6(a) and Figures 6(b)-6(c).  Figure 6(e) showed that the original CT image registered with the mathematically deformed CT image with little difference. Quantitative validation results showed that more than 90% of the voxels were within 2 mm of their intended shifts. Future work includes a further improvement to the performance of IMP method by implanting fiducial markers into the patient for further validation.

## 5. Conclusion

We developed and tested a novel method for performing deformable registration between planning and posttreatment CT images in the H&N region. The technique was able to account for tumor responses to radiation therapy by modifying image intensities of the primary tumor voxels according to the LQ model and to deal with the interpatient heterogeneity of radiobiology parameters by a scaling factor. The preliminary tests resulted in higher registration accuracy than current methods, indicating a role in H&N adaptive radiation therapy.

## Figures and Tables

**Table 1:** Statistics for Dice similarity coefficient (DSC) for the tumor using rigid registration and deformable image registration with and without the intensity-modification procedure (IMP).

Patient	DSC (rigid)	DSC (IMP)	DSC (no IMP)
1	86.7	91.6	88.0
2	78.9	95.1	82.3
3	73.5	90.9	60.7
4	86.9	96.8	86.2
5	52.5	78.2	63.4
6	71.5	89.4	53.9
7	76.8	94.0	82.5
8	79.3	95.8	75.1
9	80.4	95.2	87.5
10	76.0	93.3	80.7

Mean	76.3	92.0	76.0
